# Strength of EU-level food environment policies and priority recommendations to create healthy food environments

**DOI:** 10.1093/eurpub/ckac010

**Published:** 2022-03-09

**Authors:** Sanne K Djojosoeparto, Carlijn B M Kamphuis, Stefanie Vandevijvere, Celine Murrin, Isobel Stanley, Piotr Romaniuk, Janas M Harrington, Maartje P Poelman, the PEN Consortium

**Affiliations:** Department of Human Geography and Spatial Planning, Faculty of Geosciences, Utrecht University, Utrecht, The Netherlands; Department of Interdisciplinary Social Science, Faculty of Social and Behavioural Sciences, Utrecht University, Utrecht, The Netherlands; Sciensano, Department of Epidemiology and Public Health, Service of Lifestyle and Chronic Diseases, Brussels, Belgium; School of Public Health, Physiotherapy and Sports Science, University College Dublin, Dublin, Ireland; School of Public Health, Physiotherapy and Sports Science, University College Dublin, Dublin, Ireland; Department of Health Policy, School of Health Sciences in Bytom, Medical University of Silesia, Katowice, Poland; School of Public Health, University College Cork, Cork, Ireland; Chair group Consumption and Healthy Lifestyles, Wageningen University & Research, Wageningen, The Netherlands

## Abstract

**Background:**

Food environments impact on diets, obesity and non-communicable diseases (NCDs). Government policies are essential to create healthy food environments. This study aimed to assess the strength of European Union (EU)-level policies, and identify and prioritize actions for the EU to create healthy food environments.

**Methods:**

The Healthy Food Environment Policy Index (Food-EPI) was applied. The Food-EPI included 26 policy and 24 infrastructure support indicators. Independent experts (*n* = 31) rated the strength of EU-level policies and infrastructure support for each of these indicators (on a 5-point scale, from very weak to very strong) and identified and prioritized actions to improve food environments.

**Results:**

For 65% of the 26 policy indicators, EU-level policies were rated as weak and for 23% as very weak. For 63% of the 24 infrastructure support indicators, EU-level policies were rated as moderate and for 33% as weak. The experts recommended 18 policy and 19 infrastructure support actions to the EU. The Top 5 prioritized policy actions included three actions in the food composition domain (e.g. setting mandatory food composition targets), one action in the food prices domain and one action in the food promotion domain. The Top 5 prioritized infrastructure support actions included three actions in the leadership domain (e.g. developing a high-level NCDs Prevention Strategy) and two actions in the monitoring domain.

**Conclusions:**

There is large potential for the EU to strengthen its policies and infrastructure support in order to improve food environments. This study specifies priority actions for the EU to create healthy food environments.

## Introduction

Overweight, obesity and diet-related non-communicable diseases (NCDs) pose a major public health problem in Europe. In 2017, more than 50% of the adult population in the European Union (EU) were overweight of which 15% were obese.^1^ Unhealthy diets with excess foods containing too much sugar, saturated fat and salt (e.g. ultra-processed foods), and low in nutritious foods like fruits and vegetables, increase the risk of developing overweight, obesity and NCDs.^2^^,^^3^ According to The Global Burden of Disease Study (2019), dietary risks are among the Top 5 risks for attributable deaths in females and males.^4^

Food environments can be defined as the physical, economic, policy and sociocultural surroundings, opportunities and conditions that influence people’s food and beverage choices and nutritional status.^5^ Commercial interests have been allowed to prevail over public health in the past decades. This has resulted in ‘obesogenic’ food environments in which ultra-processed, high-fat and sugar-rich products are abundantly available and heavily marketed, much more than healthy foods.^6–8^ In European Member States, food environments often do not ensure that the healthy option is the easiest option to choose.^9^

Governments play a crucial role in reversing the obesogenic nature of food environments.^6^^,^^7^^,^^10^ Structural, ‘upstream’ government policies (e.g. marketing regulations for unhealthy foods) have the potential to support healthy diets among the entire population^10–12^ and are more likely to result in sustainable improvements in population nutrition than ‘downstream’ approaches (e.g. health mass media campaigns).^7^^,^^13^

Article 168 (1) of the Treaty on the Functioning of the EU states that a high level of human health protection shall be ensured in the definition and implementation of all Union policies and activities.^14^ Until now, predominantly food environment policies across EU Member States have been analysed and compared.^15^ A robust analysis at the supranational level is lacking. It is largely unknown to what extent the EU has implemented policies and infrastructure support that facilitate policy development and implementation to create healthy food environments. Moreover, little is known on how these policies and infrastructure support could be improved. Therefore, this study applied the Healthy Food Environment Policy Index (Food-EPI) developed by the International Network for Food and Obesity/NCDs Research, Monitoring and Action Support (INFORMAS).^10^ Globally, the Food-EPI has already been applied in more than 30 countries, mainly to evaluate national level policies.^16^ This is the first study that has adapted the Food-EPI to evaluate supranational level policies. In applying the Food-EPI tool, this study aims:

1. to assess the strength of EU-level policies and infrastructure support and identify implementation gaps.
2. to identify and prioritize policy and infrastructure support actions to improve food environments in EU Member States.

## Methods

### Study design

This mixed-methods study is conducted as part of the Policy Evaluation Network (PEN) (https://www.jpi-pen.eu/) and under the umbrella of INFORMAS (informas.org). In 2019–20, we applied the Food-EPI at EU-level.^10^ All procedures performed were in accordance with the ethical standards of the institutional committee [Science-Geosciences Ethics Review Board, Utrecht University, The Netherlands (ERB Review Geo L-19254)] and the Helsinki declaration. All participants signed an informed consent and conflict of interest form before participation.

### Study procedure

**Figure 1:**
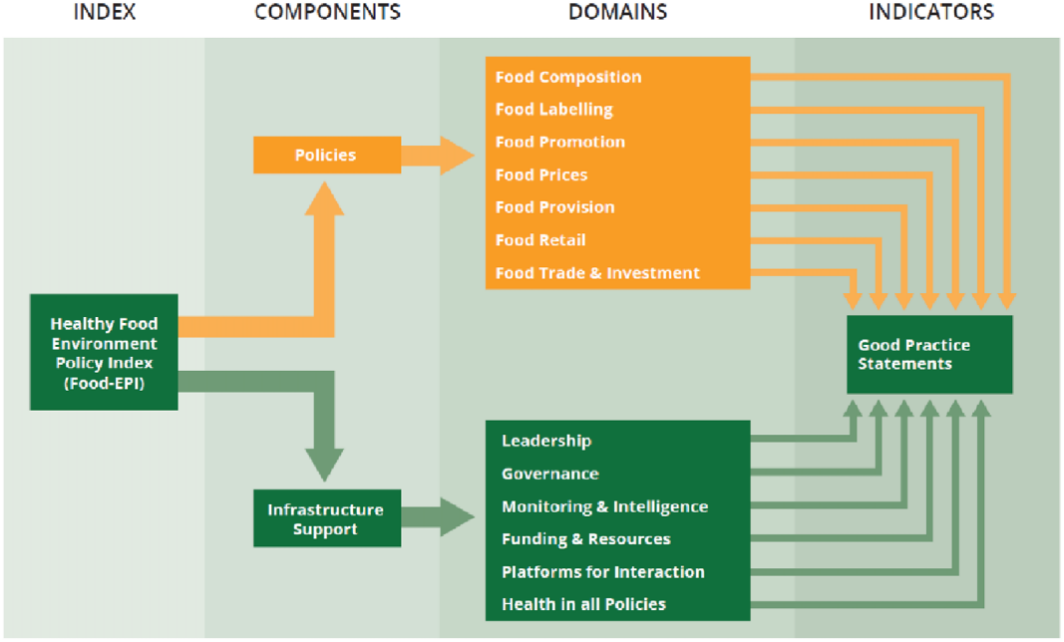
The Food-EPI, as developed by Swinburn B, Vandevijvere S, Kraak V, Sacks G, Snowdon W, Hawkes C, et al. (2013). (Food-EPI EU study, 2019–20)

The Food-EPI is an international standardized tool and process to identify important gaps in policies and infrastructure support, and to identify and prioritize future actions to improve food environments.^10^ The tool comprises indicators across seven food environment ‘policy’ domains (food composition, labelling, promotion, prices, provision, retail and trade) and six ‘infrastructure support’ domains (leadership, governance, monitoring and intelligence, funding and resources, platforms for interaction and health-in-all-policies) that support policy development and implementation to improve food environments (figure 1).^10^ There are indicators contained in each of the domains that encompass actions necessary to improve the healthiness of food environments (Supplementary file S1).

This study consisted of six steps (Supplementary file S2), which are further outlined below.

#### Step 1: tool adaptation

Before applying the Food-EPI to European countries and the EU, PEN researchers reviewed the 47 original Food-EPI indicators (February–May 2019). For each indicator, it was assessed whether the jurisdiction lies with the EU, national governments or both. In addition, as indicators were originally developed for assessing national policies, we adapted the formulation to supranational level. Furthermore, some indicators were disaggregated or added. This resulted in a total of 50 indicators included in this study, i.e. 26 policy and 24 infrastructure support indicators (Supplementary file S1).

#### Step 2: evidence document

In Step 2, evidence for EU-level policies for each of the 50 Food-EPI indicators was collected and summarized in an ‘evidence document’^17^ (February–December 2019). The Farm to Fork Strategy^9^ was not included in the evidence document, as this strategy was published after finalizing the evidence document. The evidence document was verified for completeness and accuracy by EU governmental officials working at DG SANTE, JRC, Eurostat, the OECD and EFSA.

#### Step 3: online rating survey

We conducted a workshop (February 2019) with PEN researchers to identify organizations specialized in food, nutrition, public health, obesity and diet-related chronic diseases. For each organization, we then invited one or two representatives to participate in the EU Food-EPI expert panel (November 2019–January 2020). Where specific representatives were unknown, we sent an invitation to the general e-mail addresses of the organizations. When experts declined, they could put forward a replacement. In total, 61 independent experts were invited.

In Step 3, experts were supplied with the evidence document^17^ and asked to assess the strength of EU-level policies and infrastructure support during an online survey. A total of 31 experts filled out the survey (February–March 2020), of which 29 experts fully completed and two partly. Participants rated the strength of each of the 26 policy and 24 infrastructure support indicators separately on a five-point Likert scale, indicating whether ‘The EU has put forward …’, 1 = non-existent/very weak, 2 = weak, 3 = moderate, 4 = strong or 5 = very strong policies. There was also a ‘cannot rate’ option and experts could comment on their rating. Moreover, experts were asked to formulate actions (for each policy and infrastructure domain) for the EU to create healthy food environments.

#### Steps 4–6: identification and prioritization of actions to improve food environments

Due to the 2020 Covid-19 bans on travel and meetings, face-to-face workshops with the expert panel to discuss the proposed actions, were not possible. Therefore, a different approach than outlined in the Food-EPI protocol^18^ was taken, described below in Steps 4–6.

#### Step 4: online workshops

Two online workshops were held in July 2020 with a selected group of experts, specialized in public health, nutrition or food law/politics (*n* = 3), who also had completed the online rating survey. During the workshops, all actions formulated by the entire expert panel in the online rating survey were discussed. The proposed actions were combined, narrowed down and precisely formulated. For each domain, the experts were asked whether the actions aligned with the EU competences to regulate a certain area and whether any important actions were missing.

#### Step 5a: refining actions

We made final adjustments to the action list according to the input received during the workshops. This adjusted action list was then verified by the three experts who participated in the workshops. Following this verification, the action list was sent to the full expert panel (*n* = 31) to ask whether they agreed with the actions formulated and whether any actions were missed. Final adjustments were made to the action list according to the expert panel input.

#### Step 5b: online survey to investigate which actions to recommend

The expert panel (*n* = 31) was invited for a second online survey in September 2020. A total of 16 experts participated in this survey. They were asked to indicate for each action whether they would recommend implementation of the action by the EU, using a five-point Likert scale: 1 = very much disagree, 2 = disagree, 3 = neutral, 4 = agree and 5 = very much agree.

#### Step 6: prioritization of recommended actions

In the final online survey (September–October 2020), the expert panel (*n* = 31) was asked to prioritize the recommended actions. A total of 21 experts completed this survey. Experts ranked the policy actions three times on (i) importance, (ii) achievability and (iii) equity, i.e. whether the action would lead to a reduction of socioeconomic inequalities in dietary intake. Experts ranked the infrastructure support actions twice on (i) importance and (ii) achievability. Supplementary file S3 includes a description of the three criteria. When an action was ranked as #1 it was considered to be most important, achievable or equitable.

### Data analysis

The mean score on the five-point Likert scale was calculated for each indicator to determine the strength of EU-level policies. The Gwet AC2 inter-rater reliability coefficient and its variance were determined using AgreeStat software (Agreestat 2015.6.1, Advanced Analytics, Gaithersburg, USA). For estimation of the variance, the sample of subjects to rate was set at 100% since all indicators of the Food-EPI were included for rating, while the sample of raters was set at 51% (as per the response rate of experts invited), and the finite population correction was applied (Step 3).

Regarding Step 5b, the mean score was calculated for each action based on the five-point Likert scale. Actions with a mean score of 4.0 or higher were included in Step 6.

In Step 6, we identified the highest prioritized policy and infrastructure actions by summing the ranking scores for each action. First, we calculated the scores for importance and achievability separately. Second, we calculated the total score for each action by summing the scores on importance and achievability. Sum scores could vary from 42 to 798 (policy domains) or from 42 to 756 (infrastructure support domains). A lower sum score indicated a higher perceived priority. We initially identified the Top 10 policy actions based on importance and achievability. Of this Top 10, we identified the five actions, which scored highest on equity. For the infrastructure support actions, the Top 5 was only based on importance and achievability.

## Results

### Expert panel

The 31 experts that participated in this study were working in academia, international health and food organizations, national governments, and non-governmental, professional health/food organizations and associations. The experts were specialized in food, nutrition, public health, obesity and/or diet-related chronic diseases (Supplementary file S4).

### Strength of EU-level policies and infrastructure support

**Figure 2:**
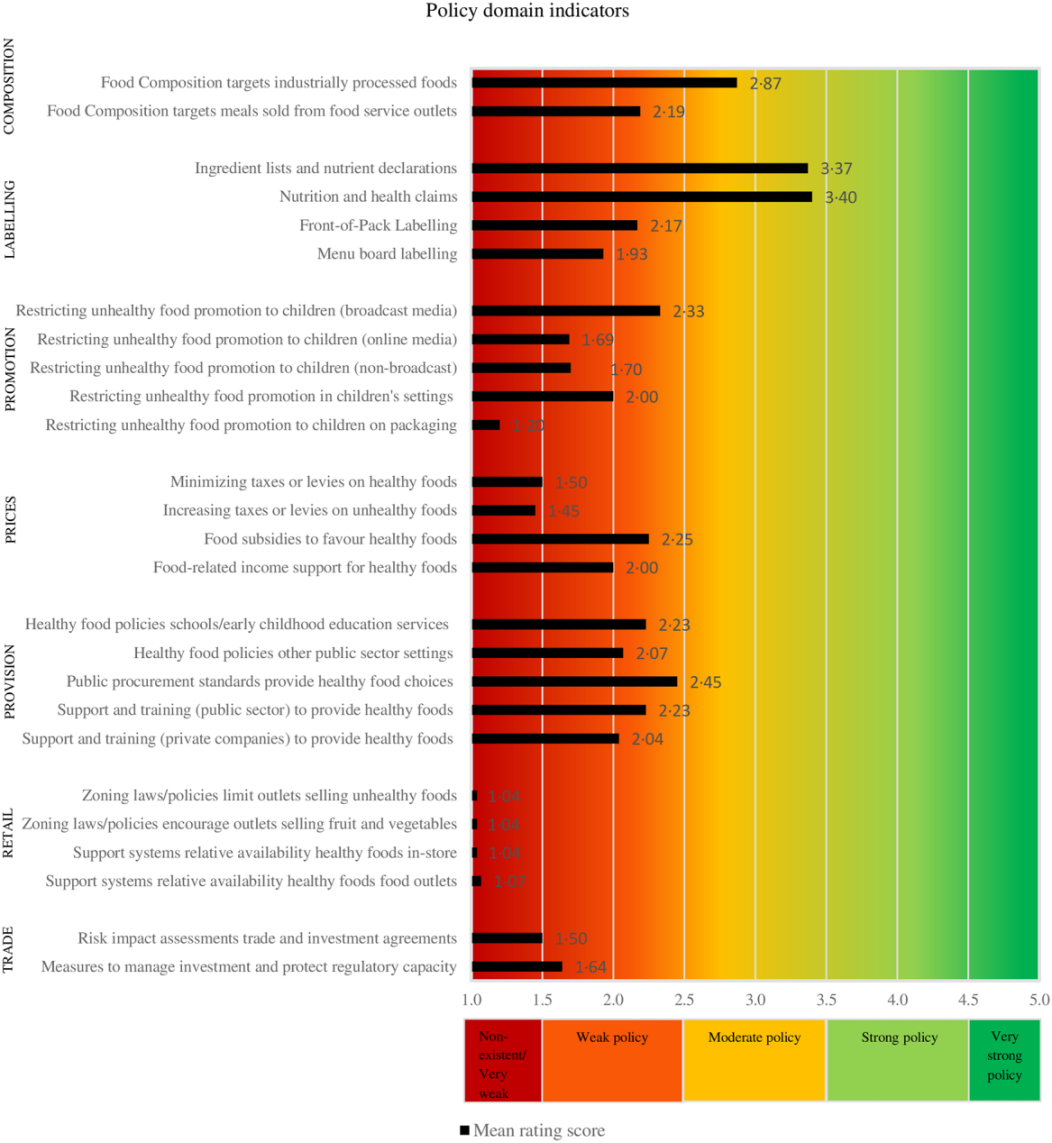
Strength of EU-level policies influencing food environments (Food-EPI EU study, 2019–20)

**Figure 3:**
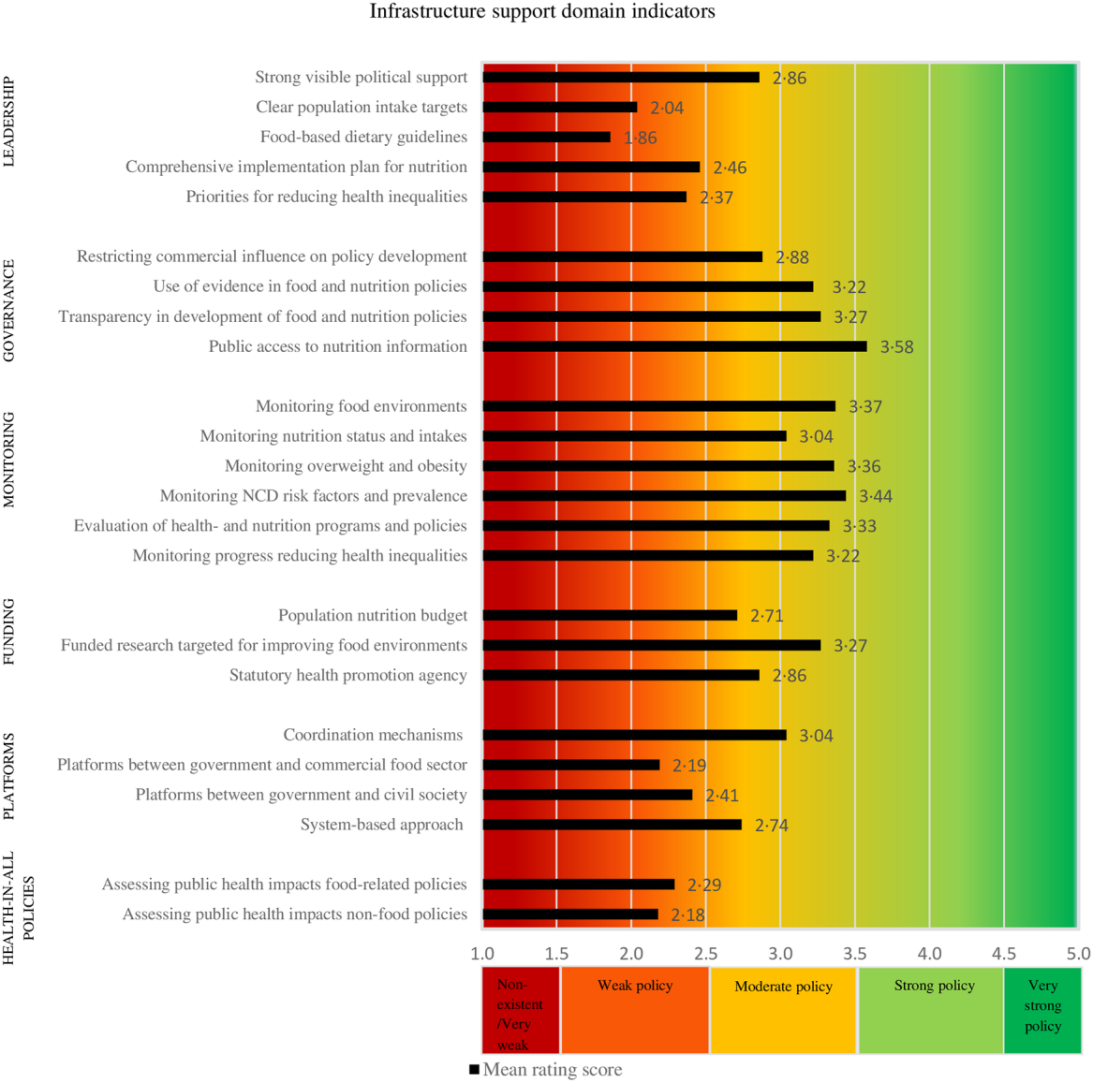
Strength of EU-level infrastructure support influencing food environments (Food-EPI EU study, 2019–20)

Figures 2 and 3 present the mean implementation score of EU-level policies and infrastructure support for each Food-EPI indicator separately, according to the experts. The Inter-rater reliability (Gwet’s AC2) for all Food-EPI indicators was 0.67 (95% CI = 0.61–0.72), which indicates that there was good agreement among experts about the strength of EU-level policies. There was more agreement on the policy indicators (Gwet’s AC2 was 0.77; 95% CI = 0.73–0.81) than on the infrastructure support indicators (Gwet’s AC2 was 0.62; 95% CI = 0.53–0.72).

#### Policy domains

The strength of EU-level policies regarding most policy indicators (17 of the 26 indicators; 65%) was rated as weak (figure 2). While the strength of EU-level policies for 6 of the 26 indicators (23%) was evenly rated as non-existent or very weak. The expert panel considered the strength of EU-level policies for 3 of the 26 indicators (12%) to be moderate.

#### Infrastructure support domains

The strength of EU-level policies regarding most infrastructure support indicators was rated as moderate (15 of the 24 indicators; 63%) (figure 3). In contrast to the policy domains, no EU-level policies with respect to the infrastructure domains were rated as very weak or non-existent. However, the EU was assessed as having weak infrastructure support for 8 of the 24 indicators (33%). Only 1 of the 24 indicators (4%) was rated as strong, namely ‘public access to nutrition information’ (‘Governance’).

### Identification and prioritization of EU-level policy and infrastructure support actions to improve food environments

Based on Steps 3 (rating survey), 4 (workshops) and 5a (refinements), 30 policy and 32 infrastructure support actions were proposed by the expert panel. In Step 5b (selection survey), 19 policy and 18 infrastructure support actions scored a 4.0 or higher and were thereby recommended to the EU to create healthy food environments in EU Member States.

#### Recommended and prioritized policy actions

The 19 policy actions recommended by the experts are detailed in Supplementary file S5. The Top 5 prioritized actions based on importance, achievability and equity (table 1) include: set mandatory, ambitious food composition targets for (i) all food categories and (ii) for processed foods and meals at quick service restaurants specifically; (iii) adopt a legislated ban for trans-fats instead of the recently introduced (2019) maximum limit of 2 g per 100 g of fat;^19^ (iv) allow Member States to implement a 0% VAT exemption on fruit and vegetables; and (v) ban marketing of unhealthy foods to children <19 years. In Supplementary file S6, the scores on importance and achievability for each action are plotted in a graph, and the five actions with the greatest potential to reduce socioeconomic inequalities in diet are indicated by a yellow shadow.

**Table 1:** Top 5 EU policy actions based on importance, achievability and equity and Top 5 EU infrastructure support actions based on importance and achievability, recommended and prioritized by the Food-EPI expert panel (Food-EPI EU study, 2019–20)

Food-EPI domain	Policy actions recommended and prioritized by the Food-EPI expert panel
Food prices	Allow Member States to implement a Value-Added Tax (VAT) exemption of 0% for all fresh fruit and vegetables, by adopting the proposal of the Commission^a^ and encourage Member States to implement this VAT exemption to encourage healthy food choices.
Food composition	Set mandatory, ambitious, comprehensive and time-specific food composition targets for added sugars, salt and saturated fat for all food categories (including processed and ultra-processed foods) sold in EU Member States (e.g. saturated fat reduction for savoury snacks of a minimum of 5% in 4 years and a minimum of an additional 5% reduction by 2026 against the individual baseline levels at the end of 2020).
Food composition	Adopt a legislated ban on trans-fats (i.e. no trans-fats are allowed instead of the maximum limit of 2 g per 100 g of fat) in processed and ultr-processed foods sold in EU Member States.
Food composition	Set mandatory, ambitious and comprehensive reformulation targets for added sugars, salt and saturated fat for processed and ultra-processed foods and meals sold at quick service restaurants (including snack food outlets) in EU Member States.
Food promotion	Introduce a new Directive [amending the Audiovisual Media Services Directive (2010/13/EU^b^)], which requires Member States to implement (i) minimum and time-based restrictions or bans on the (online) marketing of foods high in saturated fat, trans fat, salt or added sugars to children and adolescents up to 19 years old in all digital (including broadcast, online and social) media and (ii) bans on food packages for marketing foods high in saturated fat, trans fat, salt or added sugars to children and adolescents up to 19 years old.
**Food-EPI domain**	**Infrastructure support actions recommended and prioritized by the Food-EPI expert panel**
Leadership	Develop a high-level EU Non-Communicable Diseases (NCDs) Prevention Strategy.
Monitoring	Benchmark food environment policies regarding food reformulation, food labelling (incl. claims and front-of-pack labelling), food marketing, food prices, food provision in public spaces and retail (zoning laws and policies, in-store product placement) and support and coordinate the exchange of good practices between Member States (e.g. via the Open Method of Coordination).
Leadership	Include clear priorities to reduce inequalities or protect vulnerable populations in the multi-annual work programmes/annual State of the Union (e.g. by the year X we want to have reduced health inequalities in relation to diet within/between EU Member States).
Leadership	Harmonize the promotion of healthy diets with other issues of concern, such as climate change and environmental protection (e.g. showing leadership via the forthcoming eighth Environmental Action Programme and engaging with the European Environmental Agency, with its theme ‘environment and health’).
Monitoring	Recommend and support Member States to set up a monitoring system to assess the status of food environments, and to measure progress on achieving the goals of nutrition and health plans.

a EUR-Lex. Proposal for a COUNCIL DIRECTIVE amending Directive 2006/112/EC as regards rates of value-added tax COM/2018/020 final—2018/05 (CNS). https://ec.europa.eu/taxation_customs/sites/taxation/files/18012018_proposal_vat_rates_en.pdf.

b DIRECTIVE (EU) 2018/1808 OF THE EUROPEAN PARLIAMENT AND OF THE COUNCIL of 14 November 2018 amending Directive 2010/13/EU on the coordination of certain provisions laid down by law, regulation or administrative action in Member States concerning the provision of audiovisual media services (Audiovisual Media Services Directive) in view of changing market realities. EUR-Lex: https://eur-lex.europa.eu/legal-content/EN/TXT/%20PDF/?uri=CELEX:32018L1808&from=HR.

#### Recommended and prioritized infrastructure support actions

The 18 infrastructure support actions recommended by the Food-EPI expert panel are detailed in Supplementary file S7. The Top 5 prioritized actions based on importance and achievability (table 1) were: (i) develop a high-level NCDs prevention strategy; (ii) include clear priorities to reduce health inequalities in EU work programmes; (iii) harmonize the promotion of healthy foods with other issues of concern; (iv) benchmark food policies and coordinate good practices among Member States; and (v) support Member States to monitor the status of national food environments. Each infrastructure support action is plotted on importance and achievability in Supplementary file S8.

## Discussion

Overall, the experts’ ratings point to a clear need to strengthen and increase the development and implementation of EU-level food environment policies and infrastructure support. Specifically, experts rated the implementation of most food environment policy indicators as weak and most infrastructure support indicators as moderate. A total of 19 policy and 18 infrastructure support actions for the EU to create healthier food environments have been identified.

The EU performs relatively better with regard to infrastructure support than with policies directly influencing food environments, which is in line with country-level observations. An 11-country Food-EPI comparison study showed that the implementation of infrastructure support was rated higher than the implementation of food environment policies in all countries, except Chile.^20^ In the Netherlands, Ireland and Norway, implementation of infrastructure support was also rated higher than implementation of policies.^21–23^

There are a number of possible explanations for the weakness of EU-level policies directly influencing food environments. Firstly, this might be related to the competences the EU has in developing and implementing healthy food environment policies. Article 5(1) of the Treaty on the European Union^24^ states that the EU should act only when the objectives of a proposed action can be better achieved by the EU than by Member States. Article 168 (1) of the TFEU states further that EU action directed towards improving public health and preventing diseases shall complement national policies.^14^ Legislative harmonization at EU-level in the field of public health is excluded by Article 168 (5) of the TFEU, except in narrowly defined areas.^25^ Therefore, EU action in this field is mostly limited to the adoption of soft law measures such as recommendations and opinions.^25^

Secondly, national governments may have a preference to address social issues domestically rather than at EU-level.^26^ Member States may resist EU power as, in most Member States, health spending is one of the largest chunks of the national social welfare budget and citizens may expect public health policies as an expression of solidarity organized by the nation state.^27^

Thirdly, the weakness of EU-level policies might be explained by influential and dominant strategies of the food industry on governmental policies, such as lobbying and promoting industry-preferred solutions.^28–30^ Moreover, much decision-making power has been directly devolved to corporations^29^, such as the EU Platform on Diet, Physical Activity and Health, which consists of industry, NGO’s and the European Commission.^29^^,^^30^ Another example is the EU Pledge, a voluntary initiative by food and beverage companies to change advertising to children.^31^

With the Farm to Fork Strategy (2020), the EU has made positive progress in that the strategy integrates all stages of the food system (from production to consumption)^32^ and refers to the creation of a favourable food environment that makes it easier to choose healthy and sustainable diets.^8^ Some actions in the Strategy are similar to actions recommended by the experts in our study, e.g. ‘set nutrient profiles to restrict nutrition and health claims of food high in salt, sugars and/or fat’, and a ‘proposal for a harmonized mandatory front-of-pack nutrition labelling’.^9^ The Farm to Fork actions address key aspects of the food environment, although not as comprehensive as those suggested by the experts in our Food-EPI EU. Specific actions related to food promotion, retail and trade are lacking in the Farm to Fork strategy, whereas this Food-EPI EU recommends e.g. restrictions or bans on the (online) marketing of unhealthy foods to children, an EU-wide retail sector commitment to remove ultra-processed foods from near checkout counters and mandatory health impact assessments for new trade agreements. Moreover, most actions in the Farm to Fork strategy are self-regulatory, voluntary measures (e.g. expecting food companies to take action on reformulation and adapting marketing strategies), whereas this Food-EPI EU goes further and recommends that the EU develops and implements mandatory structural interventions, such as ambitious, mandatory food composition targets. However, the need for binding legislation in the form of a legislative framework for a sustainable food system has been addressed in the Farm to Fork Strategy.^9^ A recent paper outlined proposals on the scope and focus of this legislative framework, including the principle to ‘Enhance the food environments in which consumer choices are made to encourage healthy, just, affordable and sustainable outcomes’.^33^ Some EU Member States have already progressed by developing or implementing more mandatory, structural interventions, such as Denmark with a trans-fat ban^15^ and Spain with their plans to ban advertising of unhealthy foods aimed at children.^34^ This suggests that the EU could do more to commend these pioneering achievements, and encourage other Member States to better them.

Such structural policies are more likely to result in sustainable changes in food consumption.^7^^,^^35^ And as the impact of combined interventions is greater than the impact of individual interventions,^36^^,^^37^ the experts in this study emphasized that measures should be part of a high-level EU Strategy for the prevention of NCDs and recommend harmonization of the promotion of healthy diets with other issues of concern, such as environmental protection. Thus, this Food-EPI EU could be used in addition to the Farm to Fork Strategy, as the actions complement each other well in the ambition to create healthier and more sustainable food environments in EU Member States.

This study has a number of important strengths. It is the first study at EU-level that applied a comprehensive mixed-methods approach to generate insight into policy and infrastructure support gaps, as well as actions to improve food environments in the EU. Secondly, policies studied were verified by EU governmental officials and evaluated by independent experts. Thirdly, experts in this study were asked to prioritize the policy actions on equity, in addition to their importance and achievability.

Nevertheless, some limitations should be acknowledged. Firstly, due to the Covid-19 restrictions, the workshop (Step 4) was conducted online with a small group of experts instead of the face-to-face meeting with the entire expert panel. In addition, we experienced a drop-out in participation, as fewer experts participated in the follow-up surveys (*n* = 16, *n* = 21) compared to the first survey (*n* = 31), highlighting the limitations of an online format. Yet, compared to other Food-EPI studies, the number of participants in our prioritization survey (*n* = 21) was in line with other countries.^21–23^

We also have recommendations for future research. First, this Food-EPI EU constructed scorecards (figures 2 and 3) on the strength of EU-level policies, which facilitates monitoring over time. In the long-term, this study can contribute to a global database for monitoring and evaluating policies directed at improving food environments. A second recommendation is to identify ‘why’ recommended policies have or have not been successfully implemented, which can support uptake of policies.^38^ A third recommendation is to incorporate sustainability indicators in future Food-EPI studies.^16^ Fourth, it is recommended to monitor policies and practices implemented by the food industry, as a multisector response is needed in the prevention of NCDs^39^ and this could inform efforts to hold the private sector accountable.^40^ A final recommendation is to compare the outcomes of this study, with outcomes of the national Food-EPI studies conducted as part of PEN (Ireland, the Netherlands, Norway, Poland and Germany) and the H2020 Science and Technology in childhood Obesity Policy (STOP) project (Slovenia, Spain, Portugal, Estonia and Finland).

## Conclusions

Experts considered most EU-level policies directly influencing food environments in EU Member States as weak, while most infrastructure support was rated as moderate. Recommended actions should be implemented by the EU to create healthy food environments in EU Member States.

## Supplementary data

Supplementary data are available at *EURPUB* online.

## Supplementary Material

ckac010_Supplementary_Data
