# Supplementary material for: Strength of EU-level food environment policies and priority recommendations to create healthy food environments
Source: Eur J Public Health. 2022 Mar 9;32(3):504–11. doi: 10.1093/eurpub/ckac010 (PMC9159309; doi:10.1093/eurpub/ckac010)
Supplement: ckac010_Supplementary_Data [file ckac010_supplementary_data.docx]

**Supplementary files Paper “****Strength of EU-level food environment policies and priority recommendations to create healthy food environments”**

**Supplementary file 1**

There are indicators contained in each of the Food-EPI domains that encompass actions necessary to improve the healthiness of food environments and to help prevent obesity and diet-related NCDs (see Table S1). 50 good practice indicators have been included in this Food-EPI EU study.

**Table S1 Food-EPI Domains and Indicators** *[Food-EPI EU study, 2019-2020]*

| **Food-EPI Policy Domains** | |
| --- | --- |
| **Food-EPI Domain** | **Food-EPI Indicators** |
| DOMAIN 1 – FOOD COMPOSITION  Food composition targets/standards/restrictions for processed foods: This domain  concerns the extent to which the EU stimulated/proposed/developed/  implemented systems to ensure that, where practicable, processed foods minimise the energy density  and the nutrients of concern (salt, saturated fat, trans fat, added sugar). | **COMP1** Food composition targets/standards/restrictions have been established by the EU for the content of the nutrients of concern (trans fats, added sugars, salt, saturated fat) in industrially processed foods, in particular for those food groups that are major contributors to population intakes of those nutrients of concern. |
|  | **COMP2** Food composition targets/standards/restrictions have been established by the EU for the content of the nutrients of concern (trans fats, added sugars, salt, saturated fat) in meals sold from food service outlets, in particular for those food groups that are major contributors to population intakes of those nutrients of concern. |
| DOMAIN 2 – FOOD LABELLING  This domain concerns the extent to which the EU proposed/developed a regulatory system for consumer-oriented labelling on food packaging and menu boards in restaurants to  enable consumers to easily make informed food choices and to prevent misleading claims. | **LABEL1** Ingredient lists and nutrient declarations in line with Codex recommendations are present on the labels of all packaged foods. |
|  | **LABEL2** Evidence-based regulations are in place for approving and/or reviewing claims on foods, so that consumers are protected against unsubstantiated and misleading nutrition and health claims. |
|  | **LABEL3** One or more interpretive, evidence-informed front-of-pack supplementary nutrition information system(s) proposed/required by the EU, which readily allow consumers to assess a product’s healthiness, is/are applied to all packaged foods (examples are the Nutri-Score and traffic lights). |
|  | **LABEL4** A simple and clearly-visible system of labelling the menu boards of all quick service restaurants (i.e. fast food chains) is set/proposed by the EU to be implemented by the Member States, which allows consumers to interpret the nutrient quality and energy content of foods and meals on sale. |
| DOMAIN 3 – FOOD PROMOTION  This domain concerns the extent to which the EU has set/proposed policies to reduce the impact (exposure and power) of promotion of unhealthy foods to children including  adolescents across all media.  · Exposure of food marketing concerns the reach and frequency of a marketing  message. This is dependent upon the media or channels, which are used to market foods.  · The power of food marketing concerns the creative content of the marketing message. For example, using cartoons or celebrities enhances the power (or persuasiveness) of a  marketing message because such strategies are attractive to children. | **PROMO1** Effective policies are set/proposed by the EU to be implemented by the Member States to restrict exposure and power of promotion of unhealthy foods to children including adolescents through broadcast media (TV, radio). |
|  | **PROMO2** Effective policies are set/proposed by the EU to be implemented by the Member States to restrict exposure and power of promotion of unhealthy foods to children including adolescents through online and social media. |
|  | **PROMO3** Effective policies are set/proposed by the EU to be implemented by the Member States to restrict exposure and power of promotion of unhealthy foods to children including adolescents through non-broadcast media other than packaging and online/social media. |
|  | **PROMO4** Effective policies are set/proposed by the EU to be implemented by the Member States to ensure that unhealthy foods are not commercially promoted to children including adolescents in settings where children gather (e.g. preschools, schools, sport and cultural events). |
|  | **PROMO5** Effective policies are set/proposed by the EU to be implemented by the Member States to ensure that unhealthy foods are not commercially promoted to children (including adolescents) on food packages. |
| DOMAIN 4 – FOOD PRICES This domain concerns the extent to which food pricing policies (e.g., taxes and subsidies) are aligned with health outcomes by helping to make the healthy eating choices the  easier, cheaper choices. | **PRICES1** Taxes or levies on healthy foods are minimised to encourage healthy food choices (e.g. low or no sales tax, excise, value-added or import duties on fruit and vegetables). |
|  | **PRICES2** Taxes or levies on unhealthy foods (e.g. sugar-sweetened beverages, foods high in nutrients of concern) are in place and increase the retail prices of these foods by at least 10% to discourage unhealthy food choices, and these taxes are reinvested to improve population health. |
|  | **PRICES3** The intent of existing subsidies on foods, including infrastructure funding support (e.g. research and development, supporting markets or transport systems), is to favour healthy rather than unhealthy foods. |
|  | **PRICES4** The EU ensures that food-related income support programs are for healthy foods within EU countries. |
| DOMAIN 5 – FOOD PROVISION  This domain concerns the extent to which the EU ensures that there are healthy food service policies to be implemented by Member States in government-funded settings to ensure that food provision encourages healthy food choices, and the extent to which the EU actively encourages and supports private companies to implement similar | **PROV1** The EU ensures that there are clear, consistent policies (including nutrition standards) to be implemented by Member States in schools and early childhood education services for food service activities (canteens, food at events, fundraising, promotions, vending machines etc.) to provide and promote healthy food choices. |
|  | **PROV2** The EU ensures that there are clear, consistent policies to be implemented by Member States in other public sector settings for food service activities (canteens, food at events, fundraising, promotions, vending machines, etc.) to provide and promote healthy food choices. |
|  | **PROV3** The EU ensures that there are clear, consistent public procurement standards to be implemented by Member States in public sector settings for food service activities to provide and promote healthy food choices. |
|  | **PROV4** The EU ensures that there are good support and training systems to be implemented by Member States to help schools and other public sector organisations and their caterers meet the healthy food service policies and guidelines. |
|  | **PROV5** The EU actively encourages and supports private companies to provide and promote healthy foods and meals in their workplaces. |
| DOMAIN 6 – FOOD IN RETAIL  This domain concerns the extent to which the EU has the power to set/propose policies and programs to be implemented by Member States to support the availability of healthy  foods and limit the availability of unhealthy foods in communities (outlet density and locations) and in-store (product placement). | **RETAIL1** Zoning laws and policies are proposed by the EU to be implemented by the Member States to place limits on the density or placement of quick serve restaurants or other outlets selling mainly unhealthy foods in communities and/or access to these outlets (e.g. opening hours). |
|  | **RETAIL2** Zoning laws and policies are proposed by the EU to be implemented by the Member States to encourage the availability of outlets selling fresh fruit and vegetables and/or access to these outlets (e.g. opening hours, frequency i.e. for markets). |
|  | **RETAIL3** The EU ensures existing support systems are in place to be implemented by the Member States to encourage food stores to promote the in-store availability of healthy foods and to limit the in-store availability of unhealthy foods. |
|  | **RETAIL4** The EU ensures existing support systems are in place to be implemented by the Member States to encourage the promotion and availability of healthy foods in food service outlets and to discourage the promotion and availability of unhealthy foods in food service outlets. |
| DOMAIN 7 – FOOD TRADE AND INVESTMENT  This domain concerns the extent to which the EU ensures that trade and investment  agreements protect food sovereignty, favour healthy food environments, are linked with domestic health and agricultural policies in ways that are consistent with health  objectives, and do not promote unhealthy food environments. | **TRADE1** The EU undertakes risk impact assessments before and during the negotiation of trade and investment agreements, to identify, evaluate and minimize the direct and indirect negative impacts of such agreements on population nutrition and health. |
|  | **TRADE2** The EU adopts measures to manage investment and protect their regulatory capacity with respect to public health nutrition. |

| **Food-EPI Infrastructure Support Domains** | |
| --- | --- |
| **Food-EPI Domain** | **Food-EPI Indicators** |
| DOMAIN 8 – LEADERSHIP  This domain concerns the extent to which political leadership ensures that there is strong support for the vision, planning, communication, implementation and evaluation of policies and actions to create healthy food environments, improve population nutrition,  and reduce diet-related inequalities. | **LEAD1** There is strong, visible, political support (at the head of European Commission/ Parliament level) expressed at European, supra national as well as national level for improving food environments, population nutrition, diet related NCDs and their related inequalities”. |
|  | **LEAD2** Clear population intake targets have been proposed by the EU for the nutrients of concern and/or relevant food groups to meet WHO and European recommended dietary intake levels. |
|  | **LEAD3** Clear, interpretive, evidenced-informed food based dietary guidelines have been established and conveyed to EU countries. |
|  | **LEAD4** There is a comprehensive, transparent, up-to-date implementation plan linked to EU countries’ needs and priorities, to improve food environments, reduce the intake of the nutrients of concern to meet WHO and European recommended dietary intake levels, and reduce diet-related NCDS. |
|  | **LEAD5** EU priorities have been established to reduce inequalities or protect vulnerable populations in relation to diet, nutrition, obesity and NCDs |
| DOMAIN 9 – GOVERNANCE  This domain concerns the extent to which the EU has structures in place to ensure  transparency and accountability, and encourage broad community participation and inclusion when formulating and implementing policies and actions to create healthy food environments, improve population nutrition, and reduce diet-related inequalities. | **GOVER1** There are procedures in place to restrict commercial influences on the development of policies related to food environments where they have conflicts of interest with improving population nutrition. for example: restricting lobbying influences. |
|  | **GOVER2** Policies and procedures are implemented for using evidence in the development of food and nutrition policies. |
|  | **GOVER3** Policies and procedures are implemented for ensuring transparency in the development of food and nutrition policies. |
|  | **GOVER4** The EU ensures public access to comprehensive nutrition information and key documents (e.g. budget documents, annual performance reviews and health indicators) for the public. |
| DOMAIN 10 – MONITORING AND INTELLIGENCE  This domain concerns the extent to which the EU’s monitoring and intelligence systems (surveillance, evaluation, research and reporting) are comprehensive and regular enough  to assess the status of food environments, population nutrition and diet-related NCDs and their inequalities, and to measure progress on achieving the goals of nutrition and health plans. | **MONIT1** Monitoring systems, implemented by the EU, are in place to regularly monitor food environments(especially for food composition for nutrients of concern, food promotion to children, and nutritional quality of food in schools and other public sector settings), against codes/guidelines/standards/targets. |
|  | **MONIT2** There is regular monitoring of adult and childhood nutrition status and population intakes against specified intake targets or recommended daily intake levels. |
|  | **MONIT3** There is regular monitoring of adult and childhood overweight and obesity prevalence using anthropometric measurements. |
|  | **MONIT4** There is regular monitoring of the prevalence of NCD metabolic risk factors and occurrence rates (e.g. prevalence, incidence, mortality) for the main diet-related NCDs. |
|  | **MONIT5** Major programs and policies are regularly evaluated to assess their effectiveness and contributions to achieving the goals of the nutrition and health plans. |
|  | **MONIT6** Progress towards reducing health inequalities or health impacts in vulnerable populations and social and economic determinants of health are regularly monitored. |
| DOMAIN 11 – FUNDING AND RESOURCES  This domain concerns the extent to which the EU has sufficient funding invested in  ‘Population Nutrition Promotion’ (estimated from the investments in population promotion of healthy eating and healthy food environments for the prevention of obesity and diet-related NCDs, excluding all one-on-one promotion (primary-care, antenatal services, maternal and child nursing services etc.), food safety, micronutrient deficiencies  (e.g. folate fortification and undernutrition) to create healthy food environments, improved population nutrition, reductions in obesity, diet-related NCDs and their related inequalities. | **FUND1** The ‘population nutrition’ budget, as a proportion of total health spending and/or in relation to the diet-related NCD burden sufficiently contributes to reducing diet-related NCD’s. |
|  | **FUND2** EU funded research is targeted for improving food environments, reducing obesity, NCDs and their related inequalities. |
|  | **FUND3** There is a statutory health promotion agency in place that includes an objective to improve population nutrition with a secure funding stream. |
| DOMAIN 12 – PLATFORMS AND INTERACTION  This domain concerns the extent to which there are coordination platforms and  opportunities for synergies across EU departments, levels of government, and other sectors (NGOs, private sector, and academia) such that policies and actions in food and nutrition are coherent, efficient and effective in improving food environments, population nutrition, diet-related NCDs and their related inequalities. | **PLAT1** There are robust coordination mechanisms across departments and levels of government (European, national, state and local) to ensure policy coherence, alignment, and integration of food, obesity and diet-related NCD prevention policies across governments. |
|  | **PLAT2** There are formal platforms (with clearly defined mandates, roles and structures) for regular interactions between the EU and the commercial food sector on the implementation of healthy food policies and other related strategies. |
|  | **PLAT3** There are formal platforms (with clearly defined mandates, roles and structures) for regular interactions between the EU and civil society on the development, implementation and evaluation of healthy food policies and other related strategies. |
|  | **PLAT4** The governments work with a system-based approach with (local, national and European) organisations/partners/groups to improve the healthiness of food environments in EU countries. |
| DOMAIN 13 – HEALTH IN ALL POLICIES  This domain concerns the processes that are in place to ensure policy coherence  and alignment, and that population health impacts are explicitly considered in the  development of EU policies. | **HIAP1** There are processes in place to ensure that population nutrition, health outcomes and reducing health inequalities or health impacts in vulnerable populations are considered and prioritised in the development of all EU policies relating to food. |
|  | **HIAP2** There are processes e.g. Health Impact Assessment’s (HIAs) to assess and consider health impacts during the development of other non-food policies. |

**Supplementary file 2**

Figure S1. Steps of the Healthy Food Environment Policy Index 2019-2020 applied in this study (2019-2020) assessing the strength of EU policies and identifying priority actions.

*[Food-EPI EU study, 2019-2020]*


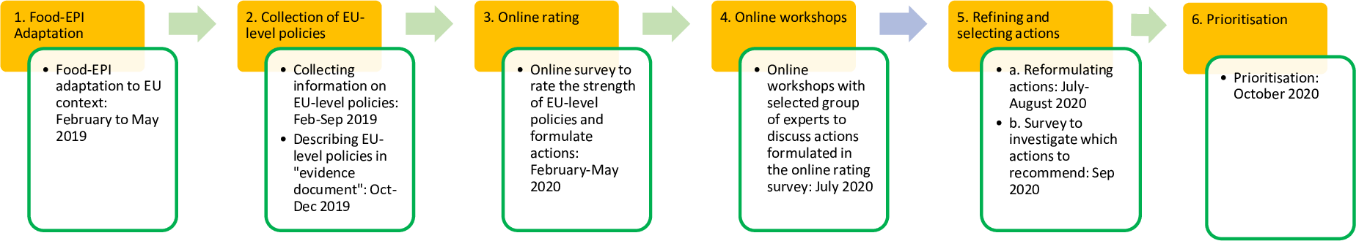


|  | |
| --- | --- |
|  |  |
|  |  |
|  |  |
|  |  |
|  |  |
|  |  |
|  |  |
|  |  |
|  |  |
|  |  |
|  |  |
|  |  |
|  |  |
|  |  |
|  |  |
|  |  |
|  |  |
|  |  |
|  |  |
|  |  |
|  |  |
|  |  |
|  |  |
|  |  |
|  |  |
|  |  |
|  |  |

|  | |
| --- | --- |
|  |  |
|  |  |
|  |  |
|  |  |
|  |  |
|  |  |
|  |  |
|  |  |
|  |  |
|  |  |
|  |  |
|  |  |
|  |  |
|  |  |
|  |  |
|  |  |
|  |  |
|  |  |
|  |  |
|  |  |
|  |  |
|  |  |
|  |  |
|  |  |
|  |  |

**Supplementary file 3. Prioritisation Criteria for Policy and Infrastructure Actions**

*[Food-EPI EU study, 2019-2020]*

| **Importance** | **Achievability** | **Equity** |
| --- | --- | --- |
| **Need**  The size of the implementation gap | **Feasibility**  How easy or hard the action is to implement | **Socio-economic effect**  Progressive/regressive effects on reducing food/diet-related inequalities |
| **Impact**  The effectiveness of the action on improving food environments and diets (including reach and effect size) | **Acceptability**  The level of support from key stakeholders including government, the public, public health and industry | **Structures vs. Individuals**  Extent to which a given policy requires environmental change rather than individual choices |
| **Other positive effects** (e.g. on protecting rights of children and consumers) | **Affordability**  The cost of implementing the action |  |
| **Other negative effects** (e.g. regressive effects on household income, infringement of personal liberties). | **Efficiency**  The cost-effectiveness of the action |  |

**Supplementary file 4: Expert panel** *[Food-EPI EU study, 2019-2020]*

| Stakeholder  Group | Expertise | Participants approached (n=61) | Participants declined (n=30) | Online rating survey (n=31) | Selection survey (n=16) | Prioritization survey (n=21) |
| --- | --- | --- | --- | --- | --- | --- |
| Academia | Academics in the field of obesity prevention, nutrition and health, food and health policies, medical science, political science, behavioural science (n=9) | 16 | No response=4  No time= 3 | 9 (of which 1 partly) | 5 | 7 |
| International health and food organizations | Representatives of international non-government health and food organizations in the field of nutrition and health promotion, diet-related chronic diseases, health and food policies (n=5) | 11 | No response=5  No time= 1 | 5 | 3 | 2 |
| Non-governmental health and nutrition organizations  (NGOs)/ associations | Representatives of non-profit organizations and (professional) associations in the field of nutrition, health, diet-related chronic diseases, health and food policies (n=10) | 22 | No response=9  No time=2  Not sufficient knowledge=1 | 10 | 6 | 8 |
| National governments/  institutes | Representatives of national governments, national/intergovernmental institutes in Europe in the field of nutrition, health and policies (n=7) | 12 | No response=3  Not sufficient knowledge=1  Not target group study=1 | 7 (of which 1 partly) | 2 | 4 |

**Supplementary file 5**

**Table S2.** **EU policy actions to improve food environments, recommended by the Food-EPI expert panel (listed in order of importance and achievability).** *[Food-EPI EU study, 2019-2020]*

| **Ranking (score)** | **Sum score**  **importance + achievability** | **Food-EPI Domain** | **Policy action recommended by the Food-EPI expert panel** |
| --- | --- | --- | --- |
| 1 | 244 | Food Labelling | Develop an EU easy-to-understand front-of-pack label (including a normative health statement) for Member States to implement for all product categories including the display on prepacked foods as well as on-shelf labelling for non-prepacked foods. |
| 2* | 294 | Food Prices | Allow Member States to implement a Value-Added Tax (VAT) exemption of 0% for all fresh fruit and vegetables, by adopting the proposal of the Commission^1^ and encourage Member States to implement this VAT exemption to encourage healthy food choices. |
| 3* | 302 | Food Composition | Set mandatory, ambitious, comprehensive and time-specific food composition targets for added sugars, salt, and saturated fat for all food categories (including processed and ultra-processed foods) sold in EU Member States (e.g. saturated fat reduction for savoury snacks of a minimum of 5% in 4 years and a minimum of an additional 5% reduction by 2026 against the individual baseline levels at the end of 2020). |
| 4 | 305 | Food Labelling | Develop and use a clear and evidence-based nutrient profiling system to prevent the use of nutrition and health claims (including function claims) on foods and meals high in saturated fat, trans fat, salt or added sugars. |
| 5* | 317 | Food Composition | Adopt a legislated ban on trans fats (i.e. no trans-fats are allowed instead of the maximum limit of 2 grams per 100 grams of fat) in processed and ultra-processed foods sold in EU Member States. |
| 6 | 341 | Food Labelling | Adjust existing regulations (e.g. food information to consumers regulation EU No 1169/2011^2^, added sugars annex^3^) to make the declaration of added or free sugars on prepacked foods mandatory. |
| 7* | 345 | Food Composition | Set mandatory, ambitious and comprehensive reformulation targets for added sugars, salt, and saturated fat for processed and ultra-processed foods and meals sold at quick service restaurants (including snack food outlets) in EU Member States. |
| 8* | 348 | Food Promotion | Introduce a new Directive, (amending the Audiovisual Media Services Directive (2010/13/EU^4^)), which requires Member States to implement (1) minimum and time-based restrictions or bans on the (online) marketing of foods high in saturated fat, trans fat, salt or added sugars to children and adolescents up to 19 years old in all digital (including broadcast, online and social) media and (2) bans on food packages for marketing foods high in saturated fat, trans fat, salt or added sugars to children and adolescents up to 19 years old. |
| 9 | 366 | Food Promotion | Ensure that the Digital Services Act creates a governance regime that enables Member States to maintain, adopt and enforce national legislation to minimize the exposure of children and adolescents up to 19 years old to foods high in saturated fat, trans fat, salt or added sugars. |
| 10 | 369 | Food Promotion | Develop and use a clear and evidence-based nutrient profiling system (e.g. such as the WHO nutrient profile model) to restrict the marketing (including online marketing) of processed and ultra-processed foods high in saturated fat, trans fat, salt or added sugars. |
| 11 | 429 | Food Promotion | Prohibit the sponsorship of foods high in saturated fat, trans fat, salt or added sugars from EU-wide sporting and other events with a legal or financial connection with the EU (e.g. events organized by the Union of European Football Associations (UEFA)). |
| 12 | 444 | Food Provision | Include minimum mandatory criteria for food procurement supporting healthy diets in schools, hospitals and public institutions, in addition to setting these criteria for sustainable food procurement as announced in the Farm to Fork Strategy. |
| 13 | 459 | Food Prices | Encourage Member States to ensure that consumer food-related income support programs distribute mainly nutritious food products supporting a healthy diet (e.g. fruit and vegetables, dietary fibre), e.g. replacing the current provision about food support in the European Social Fund Plus from ‘‘where appropriate the choice of food products to be distributed shall be made having considered their contribution to the balanced diet of the most deprived persons” to “ensure the choice of food products to be distributed is for nutritious food products supporting a healthy diet (e.g. fruit, vegetables, dietary fibre such as whole grains), and is not including foods high in trans fats, saturated fat, added sugars or salt, which contribute to a healthy diet of the most deprived persons”. |
| 14 | 471 | Food Provision | Provide food service and procurement guidelines (e.g. “the European Sustainable and Healthy Public Food Procurement guide”) to support healthy diets which also promote the role of public health dietitians/nutritionists to support public sector organizations and their caterers (e.g. by training staff, supporting the implementation of nutrition standards). |
| 15 | 474 | Food Prices | Require Member States to implement the standard VAT rate of a minimum of 15% to foods high in trans fats, saturated fat, added sugars, by adding those foods to the list of goods and services (Annex IIIa of the current proposal on VAT rates^5^) to which the standard rate of minimum 15% must always be applied. |
| 16 | 532 | Food Provision | Amend the Public Procurement Directive to include specific clauses that relate to the provision and promotion of nutritious foods supporting healthy diets in public sector settings and support the implementation by Member States via guidelines and toolkits. |
| 17 | 549 | Food in Retail | Elicit an EU-wide retail sector commitment to (1) remove ultra-processed and processed foods high in added sugars, salt, trans fat or saturated fat from near checkout counters and (2) ban (price) promotions of foods high in added sugars, salt, trans fat or saturated fat. |
| 18 | 597 | Food Trade | Make health impact assessments mandatory for new trade agreements between the EU and third countries, including explicit references to the food environment and use this evidence and information when making decisions on trade policy. |
| 19 | 604 | Food Trade | Continuously monitor the impact of trade agreements on the EU food environment, population nutrition and health (e.g. apply the European Precautionary Principle). |

- The actions are listed in order of priority considering both importance and achievability.
- The five top 10 actions based on importance and achievability, and with the highest potential to reduce dietary socioeconomic inequalities according to the experts are marked with an asterix (*)
- The top 5 policy actions based on importance, achievability and equity are marked grey.

^1^ EUR-Lex. Proposal for a COUNCIL DIRECTIVE amending Directive 2006/112/EC as regards rates of value added tax COM/2018/020 final – 2018/05 (CNS). <https://ec.europa.eu/taxation_customs/sites/taxation/files/18012018_proposal_vat_rates_en.pdf>

^2^ REGULATION (EU) No 1169/2011 OF THE EUROPEAN PARLIAMENT AND OF THE COUNCIL of 25 October 2011 on the provision of food information to consumers, amending Regulations (EC) No 1924/2006 and (EC) No 1925/2006 of the European Parliament and of the Council, and repealing Commission Directive 87/250/EEC, Council Directive 90/496/EEC, Commission Directive 1999/10/EC, Directive 2000/13/EC of the European Parliament and of the Council, Commission Directives 2002/67/EC and 2008/5/EC and Commission Regulation (EC) No 608/2004. OJ L 304, 22.11.2011, pp. 18-63. EUR-Lex: <https://eur-lex.europa.eu/LexUriServ/LexUriServ.do?uri=OJ:L:2011:304:0018:0063:EN:PDF>

^3^ European Commission, High Level Group on Nutrition and Physical Activity, 2015. Annex II, Added Sugars. EU FRAMEWORK FOR NATIONAL INITIATIVES ON SELECTED NUTRIENTS. <https://ec.europa.eu/health//sites/health/files/nutrition_physical_activity/docs/added_sugars_en.pdf>

^4^ DIRECTIVE (EU) 2018/1808 OF THE EUROPEAN PARLIAMENT AND OF THE COUNCIL of 14 November 2018 amending Directive 2010/13/EU on the coordination of certain provisions laid down by law, regulation or administrative action in Member States concerning the provision of audiovisual media services (Audiovisual Media Services Directive) in view of changing market realities. EUR-Lex: <https://eur-lex.europa.eu/legal-content/EN/TXT/%20PDF/?uri=CELEX:32018L1808&from=HR>.

^5^ European Commission. Proposal for a COUNCIL DIRECTIVE amending Directive 2006/112/EC as regards rates of value added tax (2018). <https://ec.europa.eu/taxation_customs/sites/taxation/files/18012018_proposal_vat_rates_en.pdf>

**Supplementary file 6**

**Figure S1. Importance and achievability of the 19 recommended policy actions (top 10 priority actions in green) for the EU and the five ‘green’ actions which have the greatest potential to reduce socioeconomic inequalities in diet (indicated by the yellow shadow).^[[1]](#footnote-2)^**

*[Food-EPI EU study, 2019-2020]*


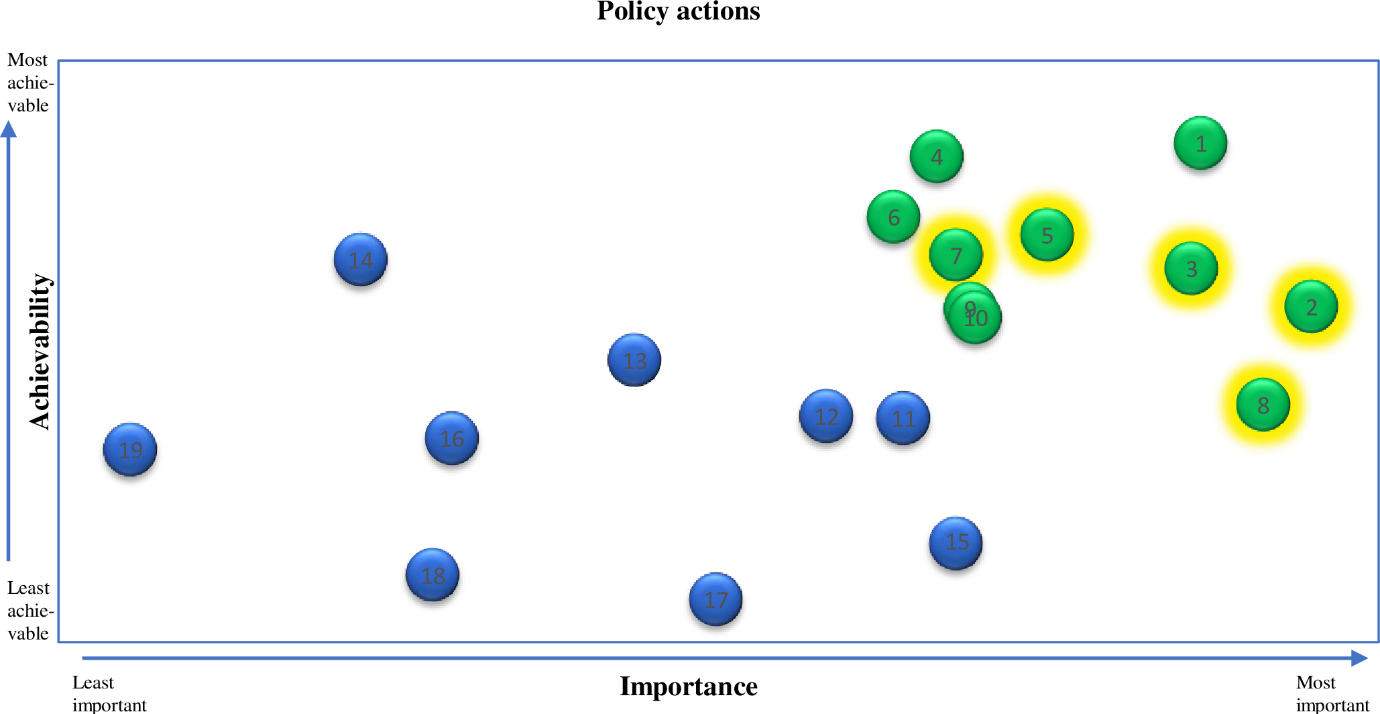


**Supplementary file 7**

**Table S3. EU infrastructure support actions, recommended by the Food-EPI expert panel (listed in order of importance and achievability).** *[Food-EPI EU study, 2019-2020]*

| **Ranking** | **Sum score**  **importance + achievability** | **Food-EPI Domain** | **Infrastructure support action recommended by the Food-EPI expert panel** |
| --- | --- | --- | --- |
| 1 | 167 | Leadership | Develop a high-level EU Non-Communicable Diseases (NCDs) Prevention Strategy |
| 2 | 210 | Monitoring | Benchmark food environment policies regarding food reformulation, food labelling (incl. claims and front-of-pack labelling), food marketing, food prices, food provision in public spaces and retail (zoning laws and policies, in-store product placement), and support and coordinate the exchange of good practices between Member States (e.g. via the Open Method of Coordination). |
| 3 | 269 | Leadership | Include clear priorities to reduce inequalities or protect vulnerable populations in the multi-annual work programmes/annual State of the Union, (e.g. by the year X we want to have reduced health inequalities in relation to diet within/between EU Member States). |
| 4 | 287 | Leadership | Harmonise the promotion of healthy diets with other issues of concern such as climate change and environmental protection (e.g. showing leadership via the forthcoming 8th Environmental Action Programme and engaging with the European Environmental Agency, with its theme 'environment and health.') |
| 5 | 302 | Monitoring | Recommend and support Member States to set up a monitoring system to assess the status of food environments, and to measure progress on achieving the goals of nutrition and health plans. |
| 6 | 306 | Leadership | Develop and adopt clear and specific population intake targets for specific nutrients (salt, added sugars, saturated fat) and specific foods (fruit and vegetables) at EU level aligned with the WHO targets and guidelines. |
| 7 | 354 | Leadership | Make diet-related health outcomes key political criteria in the European Semester and Health strand of the European Social Fund Plus (ESF+) Programme.^1,2^ |
| 8 | 359 | Governance | Develop and adopt a procedure that ensures a good balance of scientific evidence from several disciplines (e.g. economics, psychology, health science, law and consumer science) is used in the development of food and nutrition policies (e.g. secure representation from various disciplines in committees/policy boards responsible for the development of food and nutrition policies). |
| 9 | 378 | Monitoring | Evaluate food environment actions in the Member States (e.g. the recent trans-fat targets/limits in foods) by: (1) setting up an EU coordinated evaluation study of EU food environments or (2) providing funding to Member States to collect data to support this evaluation. |
| 10 | 422 | Funding and Resources | Establish an EU health promotion agency to support the design, implementation, monitoring and evaluation of actions on food environments, population nutrition and diet-related NCDs and their inequalities, e.g. such as the European Environment Agency (EEA). |
| 11 | 429 | Funding and Resources | Increase EU funded research targeting issues related to the food environment (including attention for research targeting disadvantaged groups and underrepresented household types, that are at a higher risk of NCDs and food insecurity). |
| 12 | 448 | Funding and Resources | Reallocate more Common Agricultural Policy (CAP) resources to diet-related actions targeted at consumers like the EU School Fruit and Vegetable Scheme. |
| 13 | 456 | Governance | Adopt the proposal^3^ to make the EU transparency register mandatory for lobbyists covering the Commission, Council and Parliament (including details of specific lobbying activities, e.g. when, who, what). |
| 14 | 458 | Funding and Resources | Include a heading on public health promotion in the Multiannual Financial Framework. |
| 15 | 462 | Health-in-all-Policies | Develop and adopt a health-in-all policies approach within the EU policy process and make it legally binding (by integrating health into all major EU spending programmes and setting an ambitious goal for health mainstreaming across all EU programmes, e.g. with a target of 25% of EU expenditure contributing to health objectives, as has been done with climate mainstreaming^4^). |
| ^16^ | ^477^ | Health-in-all-Policies | Establish a ‘Health in All Policies’ online portal containing at least: (1) a tracking tool providing an overview of all ongoing EU-level policy initiatives with potential impacts on health and well-being, in particular NCDs, and (2) an online directory where all impact assessments conducted for the policy initiatives identified in the first point are gathered and published. |
| 17 | 479 | Health-in-all-Policies | Make health impact assessments mandatory for all policies. |
| 18 | 577 | Health-in-all-Policies | Include diet-related health indicators when analysing health/health systems as part of the EU economic governance (the European Semester) and include health (equity) impact assessments as part of the governance-related Country Specific Recommendations of the Semester. E.g. by including diet-related outcomes as one of the indicators of the Social Scoreboard^5^ (which monitors Member States’ performance in relation to the European Pillar of Social Rights), which feeds into the preparation of the Country Reports prepared in the context of the European Semester and in the dialogue with Member States throughout the year. |

- The actions are listed in order of priority considering both importance and achievability.
- The top 5 infrastructure support actions based on importance and achievability are marked grey.

^1^ European Commission. European Social Fund. A new, stronger European Social Fund Plus. [https://ec.europa.eu/esf/main.jsp?catId=62&langId=en#:~:text=The%20European%20Social%20Fund%20Plus,the%20existing%20European%20Social%20Fund](https://ec.europa.eu/esf/main.jsp?catId=62&langId=en#:~:text=The%20European%20Social%20Fund%20Plus,the%20existing%20European%20Social%20Fund.)

^2^ ESF+ resources will be allocated to key political priorities and citizens’ concerns: ESF+ programmes and projects will have to concentrate on related challenges identified under the [European Semester](https://ec.europa.eu/info/business-economy-euro/economic-and-fiscal-policy-coordination/eu-economic-governance-monitoring-prevention-correction/european-semester_en)

^3^ European Commission. Transparency: Parliament, Commission and Council held a third round of talks on the proposal for a mandatory transparency register (2019). <https://ec.europa.eu/commission/presscorner/detail/en/STATEMENT_19_1152>

^4^ European Commission. Funding for Climate Action. Supporting climate action through the EU budget. <https://ec.europa.eu/clima/policies/budget/mainstreaming_en>

^5^ Eurostat. European pillar of social rights- indicators- social score board of indicators. <https://ec.europa.eu/eurostat/web/european-pillar-of-social-rights/indicators/social-scoreboard-indicators>

**Supplementary file 8**

**Figure S2. Importance and achievability of the 18 recommended infrastructure support actions (top 5 priority actions in green) for the EU.^[[2]](#footnote-3)^**

*[Food-EPI EU study, 2019-2020]*


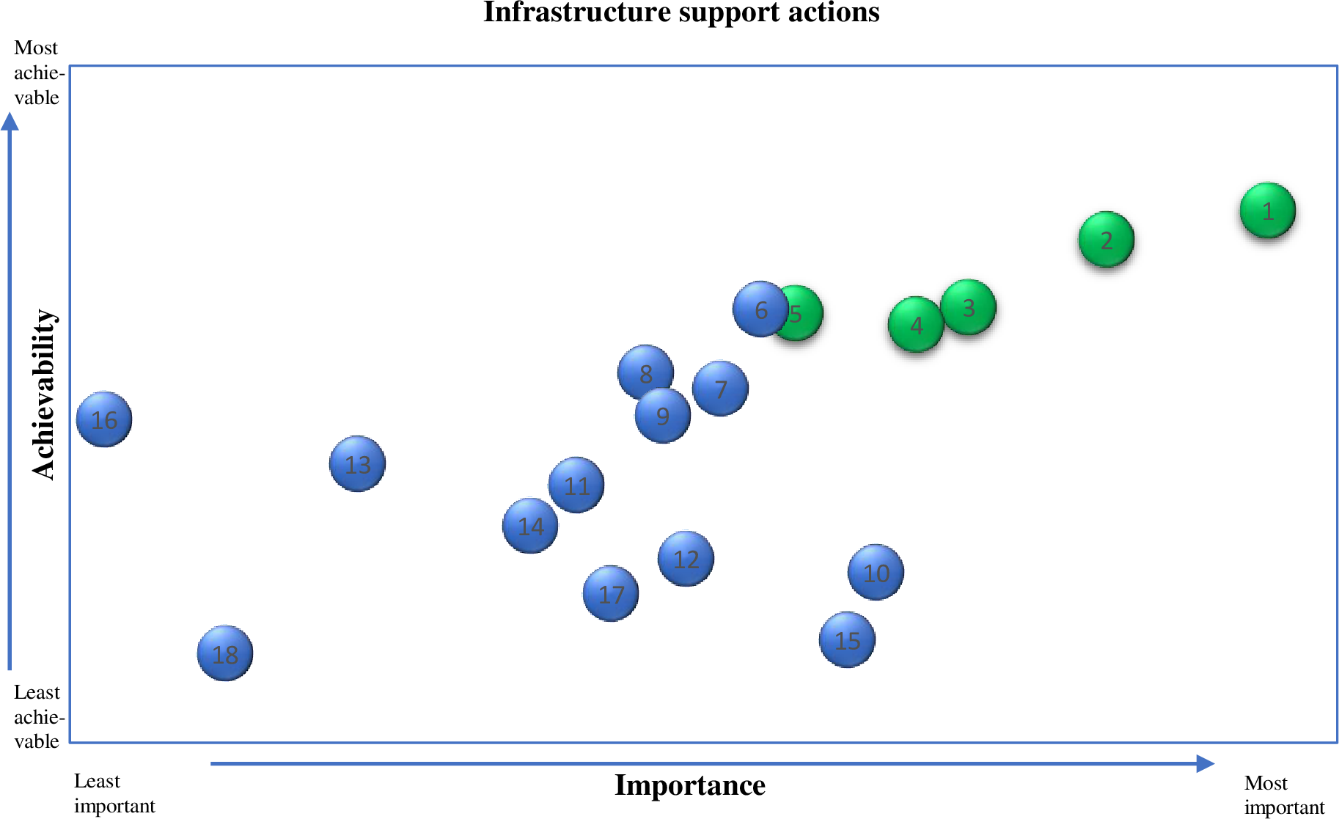


1. ^2^ The numbers of the actions (1-19) align with the numbers of the actions included in Table S2. Number 1 has the highest ranking on a combination of importance and achievability, number 19 has the lowest ranking on a combination of importance and achievability. [↑](#footnote-ref-2)
2. The numbers of the actions (1-18) align with the numbers of the actions included in Table S3. Number 1 has the highest ranking on a combination of importance and achievability, number 18 has the lowest ranking on a combination of importance and achievability. [↑](#footnote-ref-3)
